# Transmuscular quadratus lumborum (TQL) block for laparoscopic colorectal surgery: study protocol for a double-blind, prospective randomized placebo-controlled trial

**DOI:** 10.1186/s13063-020-04525-6

**Published:** 2020-06-26

**Authors:** Steve Coppens, Steffen Rex, Steffen Fieuws, Arne Neyrinck, Andre D’Hoore, Geertrui Dewinter

**Affiliations:** 1 Department of Anaesthesiology, University Hospitals of the KU Leuven, Herestraat 49, B-3000 Leuven, Belgium; 2 Department of Cardiovascular Sciences, KU Leuven-University of Leuven, Herestraat 49, B-3000 Leuven, Belgium; 3 Interuniversity Institute for Biostatistics and Statistical Bioinformatics, KU Leuven-University of Leuven & Universiteit Hasselt, Kapucijnenvoer 35, B-3000 Leuven, Belgium; 4 Department of Abdominal Surgery, KU Leuven-University Hospitals of Leuven, Herestraat 49, B-3000 Leuven, Belgium

**Keywords:** Colorectal surgery, Postoperative pain, Transmuscular quadratus lumborum block

## Abstract

**Background:**

Thoracic epidural anesthesia is no longer considered the gold standard for perioperative analgesia in laparoscopic colorectal procedures. In the search for alternatives, the efficacy of the transverse abdominal plane (TAP) block and other abdominal wall blocks such as the transmuscular quadratus lumborum (TQL) block continues to be investigated for postoperative pain management. Most of the initial studies on TAP blocks reported positive effects; however, the amount of studies with negative outcomes is increasing, most probably due to the fact that the majority of abdominal wall blocks fail to mitigate visceral pain.

The TQL block could prove attractive in the search for better postoperative pain relief after laparoscopic colorectal surgery. In several cadaveric studies of the TQL, a spread of dye into the thoracic paravertebral space, the intercostal spaces, and even the thoracic sympathetic trunk was reported.

Given the advantage of possibly reaching the thoracic paravertebral space, the potential to reach nerves transmitting visceral pain, and the possible coverage of dermatomes T4–L1, we hypothesize that the TQL provides superior postoperative analgesia for laparoscopic colorectal surgery as compared to patient-controlled intravenous analgesia with morphine alone.

**Methods and design:**

In this prospective, randomized, double-blind controlled clinical trial, 150 patients undergoing laparoscopic colorectal surgery will be included. Patients will be randomly allocated to two different analgesic strategies: a bilateral TQL with 30 ml ropivacaine 0.375% each on both sides, administered before induction of anesthesia, plus postoperative patient-controlled intravenous analgesia with morphine (TQL group, *n* = 75), or a bilateral TQL block with 30 ml saline each on both sides plus postoperative patient-controlled intravenous analgesia with morphine (placebo group, *n* = 75). Our primary outcome parameter will be the morphine consumption during the first 24 h postsurgery. Secondary endpoints include pain intensity as assessed with the numerical rating scale (NRS) for pain, time to return of intestinal function (defined as the time to first flatus and the time to the first postoperative intake of solid food), time to first mobilization, the incidence of postoperative nausea and vomiting during the first 24 h, length of stay on the post anesthesia care unit (PACU) and in the hospital, the extent of sensory block at two time points (admission to and discharge from the PACU), the doses of morphine IV as requested by the patient from the PCA pump, the total dosage of morphine administered IV, the need for and dose of rescue analgesics (ketamine, clonidine), free plasma ropivacaine levels after induction and at discharge from the PACU, and the incidence of adverse events during treatment (in particular, signs of local anesthetic systemic toxicity (LAST)).

Epidural analgesia is no longer the standard of care for postoperative analgesia in laparoscopic colorectal surgery. Until now, the most effective analgesic strategy in these patients especially in an enhanced recovery program is still unknown. Several abdominal wall blocks (TAP, fascia transversalis plane block) are known to have an analgesic effect only on somatic pain. Recognizing the importance of procedure-specific pain management, we aim to investigate whether a transmuscular quadratus lumborum block delivers superior pain control in comparison to patient-controlled intravenous analgesia with morphine alone.

**Trial registration:**

EudraCT identifier 2019-002304-40. Registered on 17 September 2019

## Administrative information

**Table:** 

Title of clinical trial	Transmuscular quadratus lumborum block (TQL) for laparoscopic colorectal surgery: A double blind, prospective randomized placebo-controlled trial.
Protocol Short Title/Acronym	TQL block for laparoscopic colorectal surgery
Study Phase if not mentioned in title	Clinical interventional trial
Sponsor name	UZ Leuven
Principal Investigator	Dr. Steve Coppens
Eudract number	2019-002304-40
Medical condition or disease under investigation	Minimally invasive laparoscopic colorectal surgery
Purpose of clinical trial	To improve pain management after laparoscopic surgery in order to minimize opioid need and enhance recovery
Primary objective	To test the efficacy of a single shot TQL block technique for laparoscopic colorectal surgery
Endpoints	Primary endpoint: Consumption of iv morphine during the first 24-h post-surgery Secondary endpoints: - Pain intensity as assessed with the numerical rating score (NRS) for pain - Requested dosage of morphine, administered via patient-controlled intravenous analgesia (PCIA) - Need for and dose of rescue analgesia - Extent of sensory block - Plasma ropivacaine levels at induction and at discharge from the PACU - Safety endpoints: Incidence of adverse events (local anesthetic systemic toxicity (LAST), nausea and vomiting, lasting sensory or motoric block, needling hematoma) - Time to first bowel movement and food intake
Sample Size	150 patients (1:1 allocation ropivacaine vs placebo)
Summary of eligibility criteria	- 18–75 years of age - BMI ≤ 35 - Patient is able to give informed consent - Patient understands the use of morphine PCIA - Patient is scheduled for elective colorectal surgery - ASA I- III - Patient has no inflammatory bowel disease with chronic pain treatment.
IMP, dosage and route of administration	Ropi-group: The total dose of the local anesthetic ropivacaine will be 225 mg in a volume of 60 ml. Bilateral administration of 30 ml ropivacaine 0,375% each using the transmuscular approach to the deep quadratus lumborum fascial layer.
Comparator product(s)	Placebo-group: 30 ml normal saline 0,9% each will be injected bilaterally.
Maximum duration of treatment of a subject	The TQL block will be placed pre-operatively. Follow up till day of discharge
Version and date of final protocol	SC 06 – 23-04-2020

## Background

Locoregional anesthesia has become a key element in multimodal analgesia [1].

With the increasing acceptance of and evidence for enhanced recovery protocols, the use of regional anesthesia continues to broaden. While the use of thoracic epidural anesthesia cannot be considered the gold standard anymore in laparoscopic colorectal procedures, the efficacy of the transverse abdominal plane (TAP) block and other abdominal wall blocks remains to be established [2, 3]. Though most of the initial studies on TAP blocks demonstrated at least some beneficial effects, the amount of studies with negative outcomes is increasing [4]. Most of the abdominal wall blocks, like TAP and fascia transversalis plane block, probably only affect somatic (i.e., abdominal wall) pain and most likely do not permit spread of local anesthetics to the paravertebral space which would allow the anesthetics to reach nerves transmitting visceral pain. Recently, we conducted a study on the efficacy of the fascia transversalis block (misnamed QLB 1) in colorectal surgery and were unable to find any significant improvement with respect to postoperative pain and opioid consumption [5].

We think that the efficacy of these blocks has been waning in the last years primarily because of advancements in surgery. Increasing experience in laparoscopic surgery with low flow/low pressure pneumoperitoneum has probably decreased the severity of somatic wall pain [6]. In contrast, visceral pain has largely remained unaffected by the new techniques and is still most effectively treated with opioids.

Notably, visceral pain stimuli are transmitted via the ventral branches of the spinal nerves. Therefore, the TQL block (first described by Borglum et al.) could prove beneficial in the search for better postoperative outcomes [7].

**Fig. 1:**
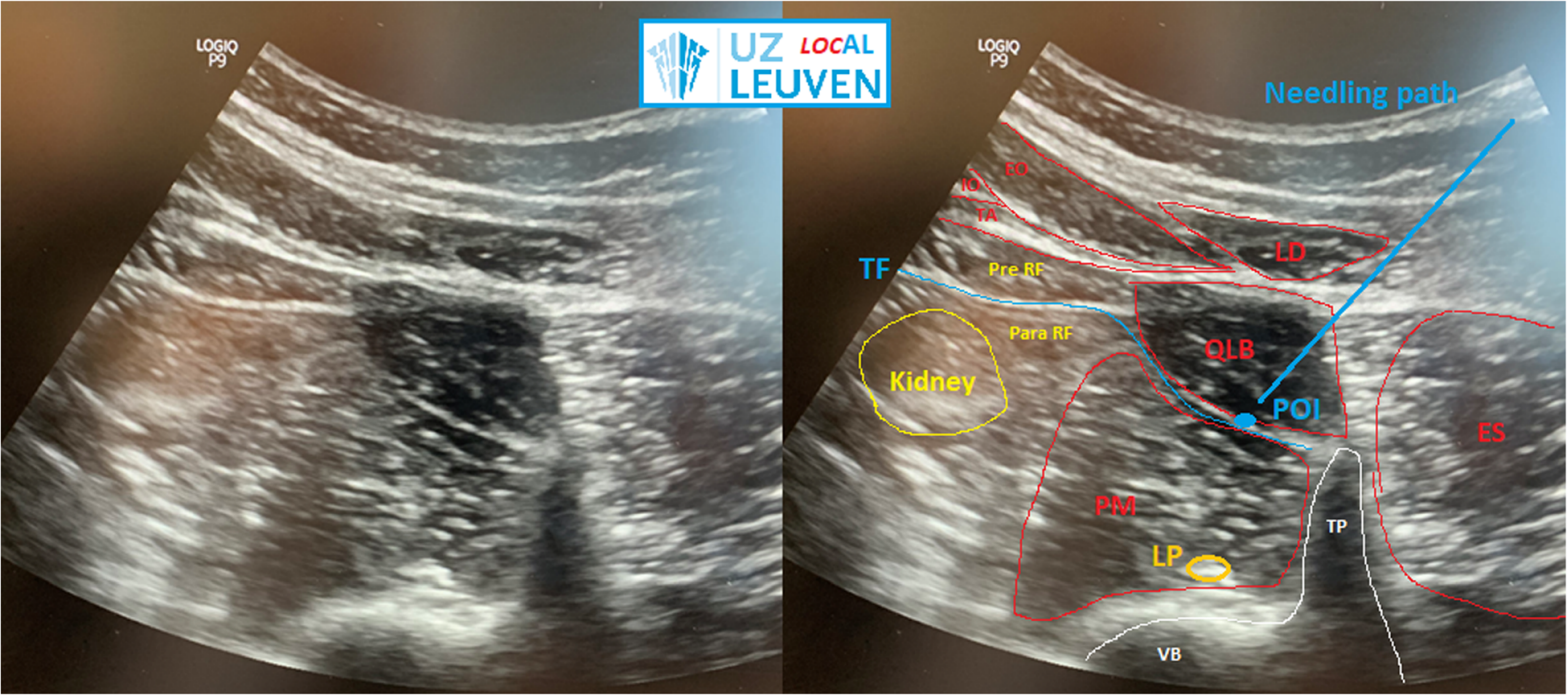
TQL sonoanatomy. Ultrasound image of UZ Leuven LOCAL (LOCoregional Anesthesia Leuven) depicting sonoanatomy, needle target, and point of injection. QLB, quadratus lumborum muscle; EO, external oblique muscle; IO, internal oblique muscle; TA, transverse abdominal muscle; LD, latissimus dorsi muscle; ES, erector spinae muscle group; PM, psoas major muscle; TF, transversalis fascia; VB, vertebral body; TP, transverse process; Kidney and Pre RF, pre-renal fat; Para RF, pararenal fat; LP, lumbar plexus in the psoas major; POI, point of injection with needling path

With this block, a spread of local anesthetics is achieved between the psoas major (PM) muscle and the quadratus lumborum (QL) muscle. Controversy on the exact anatomy remains. Some claim the anterior thoracolumbar fascia is the main target at that site. Most likely, the fascial interspace posterior to the transversalis fascia between PM and QL is the real point of injection. There is no evidence of an anterior thoracolumbar fascia, and the posterior muscle groups like the erector spinae group (iliocostalis muscle, longissimus muscle, and spinalis) also have a different origin of embryonic development (Fig. 1).

In this compartment, the ventral rami of the spinal nerves pass by at the dorsal side of the quadratus lumborum muscle. Because TQL remains a fascial plane wall block and as such depends on an extensive spread of local anesthetics to be effective, this field block requires a relatively high volume of local anesthetics (20–30 ml) due to the distance between injection site and target area.

The TQL has been shown to provide adequate analgesia in several smaller studies and case reports [8, 9]. However, to the best of our knowledge, there is no published data yet on TQL in a randomized, double-blind, placebo-controlled prospective study in colorectal surgery. There is a current RCT still ongoing on this very same subject (EudraCT number: 2017-005200-96). Analgesia after a TQL is thought to be achieved by the paravertebral and craniocaudal spread of local anesthetics and through the coverage of the lateral cutaneous branches of the thoracoabdominal nerves T1–T12 (ventral rami) as in a posterior TAP. In several cadaveric studies of the transmuscular quadratus lumborum block, spread of dye into the thoracic paravertebral space, the intercostal spaces surrounding somatic nerves, and even the thoracic sympathetic trunk was reported [10, 11]. Another cadaver study specifically compared the spread of dye when using a fascia transversalis block (misnamed QLB1), anterior quadratus lumborum block (misnamed QLB2), or TQL block. In this paper, however, the authors failed to find spread to the thoracic paravertebral area after TQL. The work by Carlin et al. however was severely criticized and even rejected in previous quoted cadaver study [10]. Given the advantage of probably reaching the thoracic paravertebral space, the potential to reach nerves transmitting visceral pain, and the possible coverage of dermatomes T4–L1, we hypothesize that the TQL provides superior postoperative analgesia for laparoscopic colorectal surgery in comparison to patient-controlled intravenous analgesia with morphine alone.

## Methods and design

### Study design

This study is a single-center, prospective, randomized, double-blind, controlled trial.

### Study aim

The aim of this trial will be to investigate the efficacy of the TQL block on postoperative pain and enhanced recovery.

### Study registration

The trial will be carried out in compliance with the principles of the Declaration of Helsinki, the principles of Good Clinical Practice, and following all regulatory requirements. The study is approved by the ethics committee of the University Hospitals Leuven on 17 September 2019 with the reference number S62905. B, the Clinical Trials Centre of the University Hospitals Leuven, and the “Federaal Agentschap voor Geneesmiddelen en Gezondheidsproducten.” The study is registered in the European Clinical Trials Database of the European Medicines Agency, EudraCT number: 2019-002304-40, 17 September 2019.

**Fig. 2:**
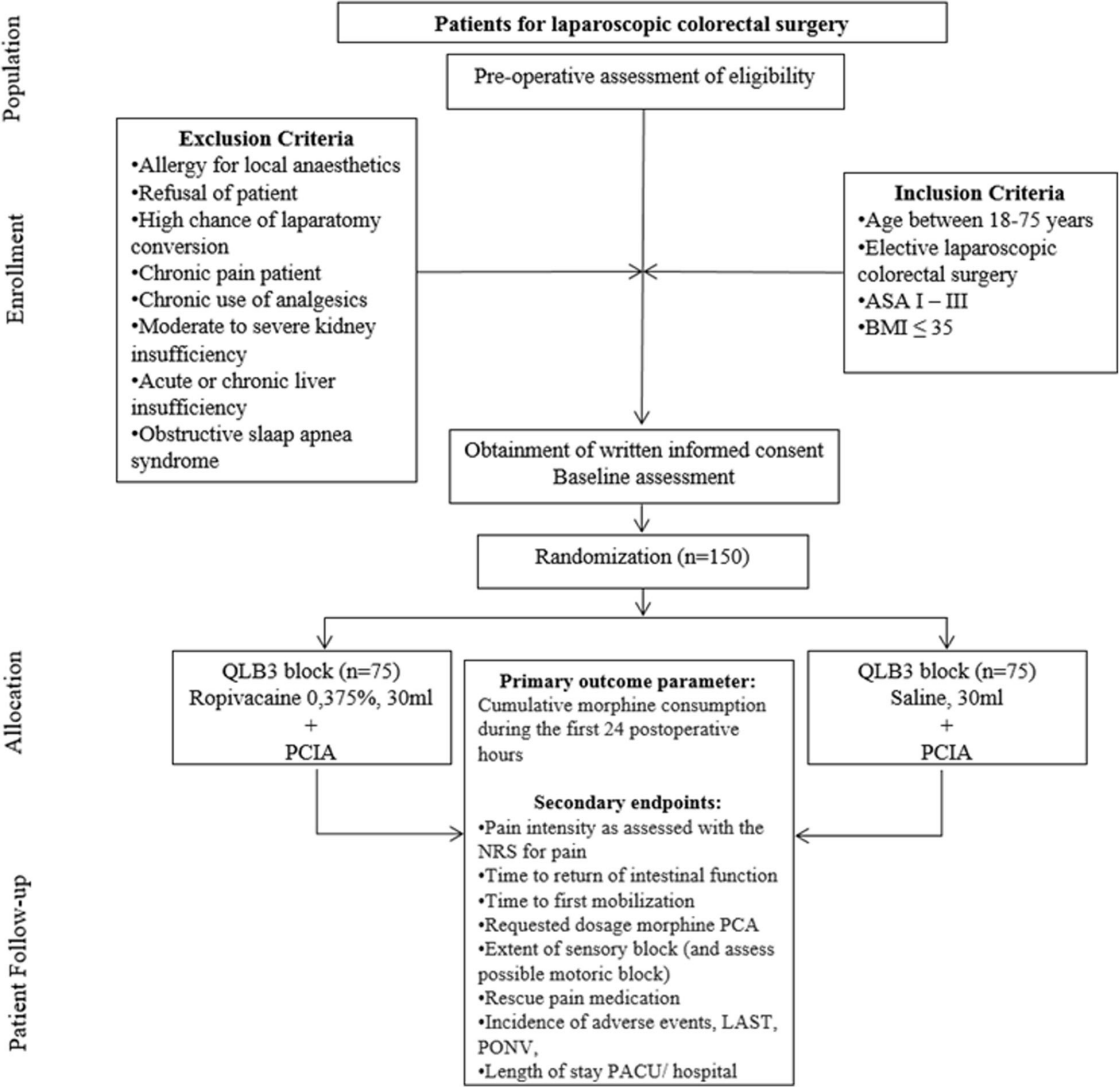
Flow diagram of the study. ASA, American Society of Anesthesiologists classification of physical status; BMI, body mass index; TQL (QLB3), transmuscular quadratus lumborum block; PCIA, patient-controlled intravenous analgesia; NRS, numeric rating score; LAST, local anesthetic systemic toxicity; PACU, postanesthesia care unit

Protocol amendments if needed will be re-sent to the ethical committee for revision. Any amendments will be made by the PI, and protocol changes accepted by the committee will be adjusted accordingly. Any breach of protocol during the recruitment and trial phase will be fully documented using a breach report form. Amendments will be added to clinical trial registries (Fig. 2).

### Recruitment

All consecutive patients planned for elective laparoscopic colorectal surgery in an enhanced recovery program will be included in the trial after obtaining informed consent. Patients will be recruited in the Department of Abdominal Surgery of the University Hospitals of the KU Leuven, Belgium. Possible risks will be explained to the patients. Patients willing to participate in the study will get information bedside, and written informed consent will be obtained. Informed consent will be the responsibility of the principal investigator (PI) or the study nurse. According to the Good Clinical Practice guidelines, all information will be given to the patient and family either at the ward 1 day before surgery or during the preoperative counseling. Two informed consents will be signed by both parties. One is securely stored and labeled in the clinical research facility of the Department of Anaesthesiology UZ Leuven, Belgium. Patients will receive the second informed consent after recruitment. Recruitment can of course always be retracted before, during, or even after trial start and will have no influence on further treatment of patient.

On the consent form, participants will be asked if they agree to use of their data should they choose to withdraw from the trial. Participants will also be asked for permission for the research team to share relevant data with people from the Universities taking part in the research or from regulatory authorities, where relevant. This trial involves collecting biological specimens for storage. Storage of specimens will be anonymized, coded, and stored in the UZ Leuven Clinical Trials Centre. Secondary studies or analysis is not planned on biological specimens.

There is no anticipated harm and compensation for trial participation. No provision for post-trial care is foreseen. Patients however have the benefit of a trial insurance protection against potential harm by the UZ Leuven, which is provided and stated in the informed consent.

### Randomization

Patients will be randomized to one of the 2 study groups. Patients in the TQL group will receive a bilateral TQL block with 30 ml ropivacaine 0.375% on both sides (placed before the induction of anesthesia) plus postoperative patient-controlled intravenous analgesia (PCIA) with morphine (TQL group, *n* = 75). Patients in the placebo group (P-group) will receive a bilateral TQL block with 30 ml saline 0.9% on both sides (placed before the induction of anesthesia) plus postoperative PCIA with morphine (P-group, *n* = 75). Patients will be randomized using a computer-generated permuted block randomization sequence (variable block size, 1:1 allocation). Allocation concealment will be ensured by enclosing assignments in sealed, opaque, sequentially numbered envelopes which will be opened only upon arrival of the patient in the preparatory block rooms [12]. An independent anesthesiologist will prepare the trial medication while patients and the attending anesthesiologists will be blinded. Code-break will only be allowed if the patients show life-threatening symptoms of LAST to allow appropriate treatment. Postoperative outcomes will be assessed by research personnel that remains blinded to the type of intervention throughout the study.

### Inclusion and exclusion criteria

Inclusion criteria are as follows: (1) age between 18 and 75 years, (2) patient scheduled for elective laparoscopic colorectal surgery, (3) American Society of Anesthesiologists classification of physical status < IV, (4) body mass index (BMI) ≤ 35, and (5) patient able to understand the use of intravenous patient-controlled anesthesia. The exclusion criteria are as follows: (1) refusal of the patient, (2) known hypersensitivity to any study medication, (3) chronic opioid use or chronic pain patient, (4) liver insufficiency (defined as a serum bilirubin ≥ 34 μmol/l, albumin ≤ 35 g/dl, INR ≥ 1.7), (5) renal insufficiency (defined as a glomerular filtration rate < 44 ml/min), (6) morbid obesity (defined as a BMI > 35), (7) obstructive sleep apnea syndrome, and (8) inability to operate a PCIA system.

### Interventional plan

**Fig. 3:**
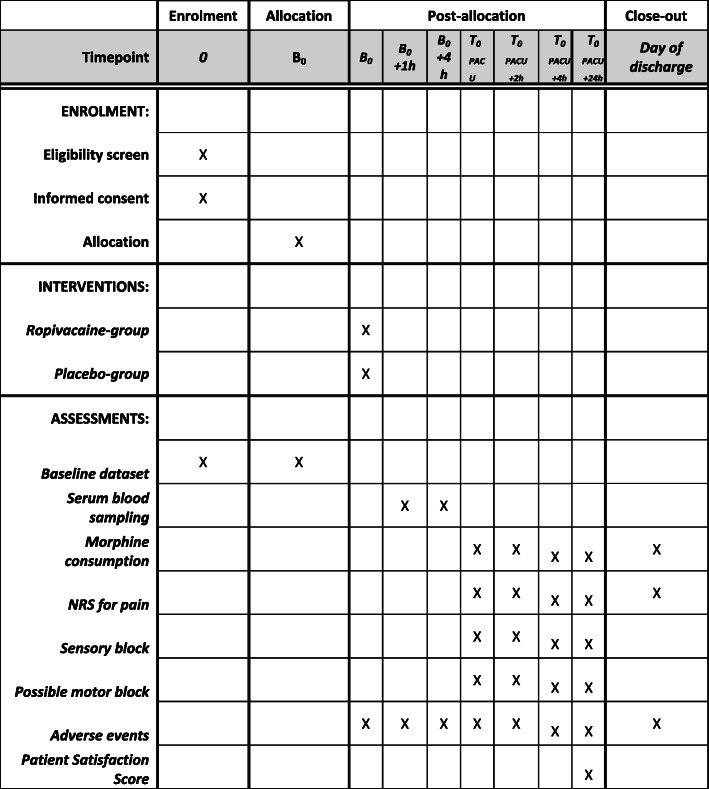
SPIRIT figure. Standard Protocol Items: Recommendations for Interventional Trials (SPIRIT). h, hours; d, days; NRS, numerical rating scale; VARC, Valve Academic Research Consortium

The ERAS (“Enhanced Recovery After Surgery”) protocol will be used in all patients in order to standardize perioperative treatment in both groups [13]. The ERAS protocol includes the following: (1) no preoperative bowel preparation, (2) avoidance of prolonged fasting, (3) no premedication, (4) intraoperative administration of PONV and antibiotic prophylaxis, (5) maintenance of normothermia, (6) restrictive fluid management, (7) early postoperative removal of the gastric tube and the bladder catheter, (8) early oral nutrition, and (9) early mobilization of the patient (Fig. 3).

### Induction and maintenance of anesthesia

In general, management will be performed according to our institutional standards. It is, however, possible that the attending anesthesiologist changes this management to optimize the patients’ care.

Prior to anesthesia, patients must be in a fasting state for 6 h and no premedication is given. After application of a 5-lead electrocardiogram and pulse oximetry, a peripheral intravenous line (16-gauge cannula) and a radial arterial catheter (20-gauge) for the invasive measurement of arterial blood pressure will be placed. After preoxygenation (FiO_2_ = 1.0), general anesthesia will be induced with a combination of an intravenous infusion of remifentanil (0.5 μg kg^−1^ min^−1^) followed by a bolus of propofol 0.5–1 mg kg^−1^. Tracheal intubation will be facilitated by a bolus administration of rocuronium 0.6 mg kg^−1^. Standard American Society of Anesthesiologists monitoring will be completed with temperature and capnography measurements. In addition, the bispectral index (BIS) will be used to standardize depth of anesthesia.

Further curarization will be left upon the discretion of the attending anesthesiologist and according to the results of relaxometry. PONV prophylaxis will be achieved with 0.1 mg kg^−1^ (max. 4 mg) intravenous dexamethasone at induction and 0.1 mg kg^−1^ (max. 4 mg) intravenous ondansetron 30 min before the end of surgery.

General anesthesia will be maintained with sevoflurane 1.5–2.0% (FiO_2_ = 0.4–0.5), titrated to achieve a BIS of 40–60. Analgesia is achieved with a continuous infusion of remifentanil (0.1–0.3 μg kg^−1^ min^−1^) and adjusted depending on patients’ reactions, spontaneous movements, sweating, and/or sudden increase in heart rate or arterial pressure.

### Interventional treatment

#### TQL group

Before induction of anesthesia, the patient is placed in a left and right lateral decubitus position. A low-frequency 18–6 MHz curvilinear ultrasound transducer is placed just above the anterior and posterior iliac crest and well below the rib cage. The transverse process (TP), the full vertebral body (VB), and the 3 important muscles making up the “shamrock” sign (the erector spinae muscle at the back (ESM), the QL muscle itself, and the psoas muscle (PM)) will be identified [14].

The interfascial space between the QL muscle and the PM will be our main target. Aligning the probe more cephalad along the midaxillary line can improve visualization; however, the space between iliac crest and rib cage can be minimal. There are 3 possible approaches described thus far.

**Fig. 4:**
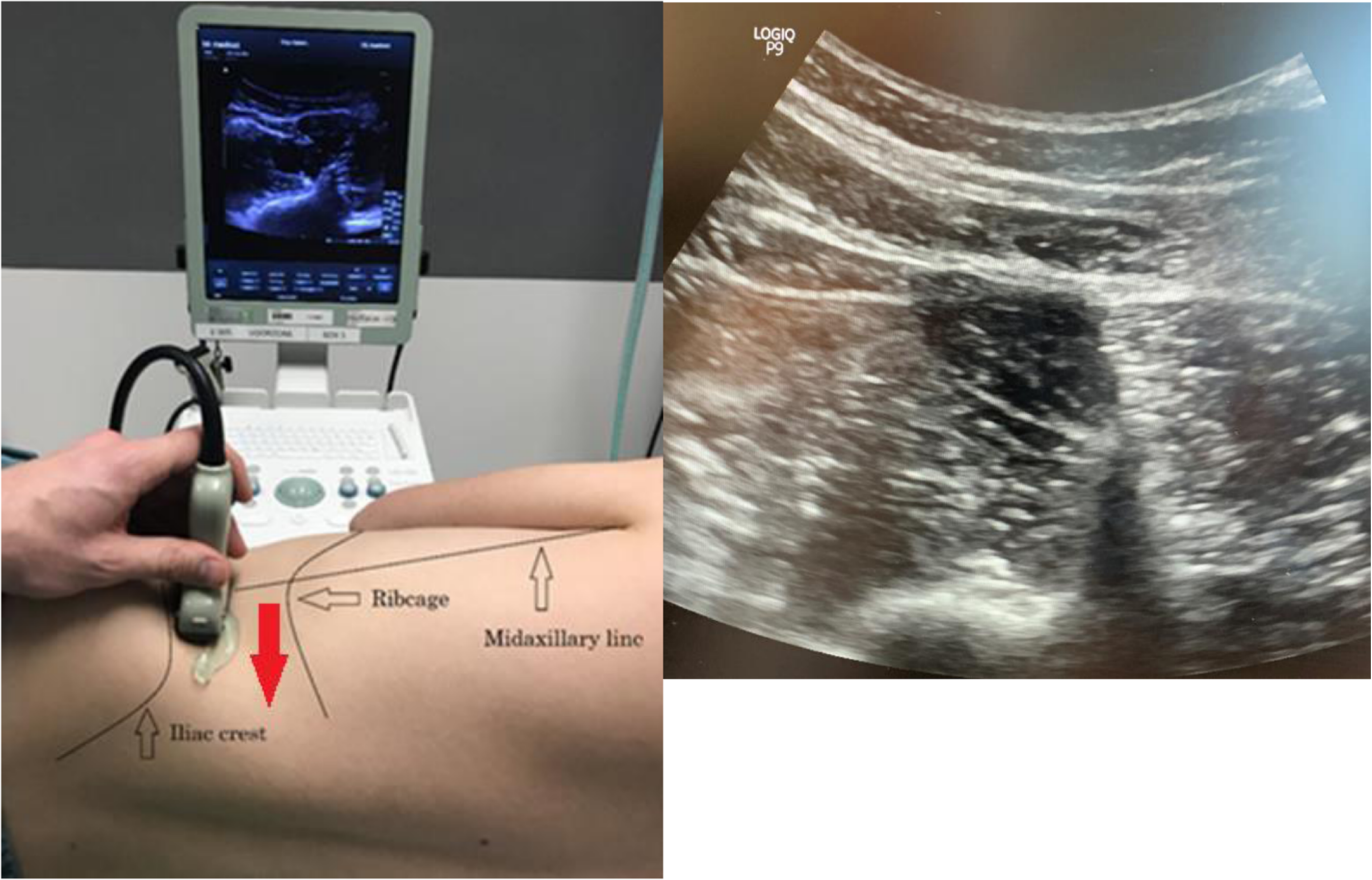
US-guided anatomy of the TQL. Alignment and ultrasound image of the TQL using 3 steps identifying first abdominal wall muscles anterior then sliding to posterior “shamrock” sign and movement of pre- and pararenal fat

All are transmuscular targeting the fascial interspace between psoas major and quadratus lumborum muscle: the classical posterior approach, the more cephalad subcostal approach, and the posteromedial approach (transverse oblique paramedian, or TOP). Depending on the patient’s anatomy, we will perform a classical posterior technique, only reverting to the TOP procedure if this improves visualization (Fig. 4).

Correct identification of the point of injection will follow a well-defined and recently published 3-step method [15]:

- Identifying the abdominal wall muscles anterior (internal oblique, external oblique, transverse abdominal)
- Identifying the so-called shamrock sign
- Clearly visualizing moving tissue to distinguish the fascia transversalis from the tissue interspace between psoas major and quadratus lumborum muscle (the two muscles do not move during respiration)

#### Placebo group

Patients in the placebo group will receive a bilateral placebo block bolus using 30 ml normal saline 0.9%.

To achieve blinding of the patients and study observers, the trial medications have the same volume of 30 ml and are identically looking. Both the investigational medicinal product and placebo will be labeled with the mark “trial medication.”

### Postoperative analgesia

Irrespective of group allocation, postoperative analgesia will be provided by administering acetaminophen IV (15 mg/kg 4/day), ketorolac 10 mg IV (20 mg over 24 h), and a bolus of 0.1 mg/kg of morphine at the end of surgery. Following surgery, patients will receive a morphine IV PCA pump which will be programmed in an on-demand-only mode. The PCIA pump will be set at 1.5 mg every 7 min with a maximum of 30 mg every 4 h.

Further analgesic treatment is dependent on the protocol used on the ward with intravenous acetaminophen (15 mg/kg 4/day) and tramadol-hydrochloride (50 mg 4/day) being used in a fixed scheme.

### Postoperative care unit

The patients will be transferred to the PACU for continuous monitoring of vital signs and the Aldrete score. In the PACU, severity of pain will be assessed at rest and during coughing by a numeric rating scale (NRS) (0 = no pain, 10 = the worst imaginable pain). In case of severe surgery-related pain despite the adequate use of the morphine IV PCA, an extra bolus of morphine can be given IV until adequate pain levels are reached (1–2 mg IV up to 0.1–0.2 mg/kg). In case of persistent pain, and NRS ≥ 3, rescue medication will be initiated. Ketamine will be the first drug of choice administered using boli of 0.1 mg/kg IV. In case of an insufficient effect and a persistent NRS ≥ 3, a bolus of clonidine 2 μg/kg can be given IV. Severity of pain will be monitored every 15 min during the first 2 h of the PACU stay and hourly during the remaining PACU stay.

PONV will be treated with iv-dehydrobenzperidol 0.625 mg (PACU) or iv-ondansetron 4 mg (on the ward). Patients will stay at least 4 h in the PACU. Patients will be discharged from the PACU only once the Aldrete score is 9 and once there is no evidence for pain and/or PONV.

### Laboratory tests

Serum-free ropivacaine will be sampled at 1 h after injection and at 4 h postinjection.

Storage of specimens will be anonymized, coded, and stored in the UZ Leuven Clinical Trials Centre. Secondary studies or analysis is not planned on biological specimens. All samples will be analyzed in batch at the conclusion of the trial. Storage is provided in a secured clinical trial deep-freeze facility (Panasonic Biomedical equipment VIP series) at strict temperature control of -80 degrees Celcius. Quantitative Mass Spectrometric Analysis (QMAs) of ropivacaine on all serum samples will be performed by a specialized and highly experienced laboratory affiliated to the UZ Leuven. All samples are transported and remain coded and anonymized. The aforementioned laboratory is independent, has no other involvement, and is not a stakeholder in the study.

### Follow-up visits

Patients will be visited once daily throughout their hospital stay by research personnel.

Twenty-four hours after surgery, the morphine IV PCA system will be removed and data will be extracted (number of morphine doses demanded by the patient, number of morphine doses delivered by the PCA pump).

### Primary endpoint

As the primary outcome parameter, we will evaluate the cumulative morphine consumption in the first 24 postoperative hours.

### Secondary endpoints

The secondary outcome parameters include following: (1) pain intensity as assessed with the numerical rating score (NRS) for pain, (2) time to first mobilization, (3) the incidence of postoperative nausea and vomiting during the first 24 h, (4) length of stay on the PACU and in the hospital, (5) extent of sensory block at two time points, (6) number of morphine boli as requested by the patient from the PCIA system, (7) total dosage of morphine IV delivered, and (8) the need for and dose of rescue analgesia (ketamine, clonidine).

### Safety endpoints

Safety endpoints measured will include the incidence of (serious) adverse events, the occurrence of signs of local anesthetic systemic toxicity (LAST), and the plasma levels of free ropivacaine.

### Trial safety

The study medication will be administered to patients with standard hemodynamic monitoring in the setting of a fully equipped operation theater. The administration of the trial medication will be stopped immediately in case that the patient shows any adverse event during the procedure. Also after leaving the operation theater, all patients will still be meticulously monitored for the appearance of eventual (severe) adverse events, first on the PACU, later on the surgical ward. In addition, the inclusion of each individual patient into the trial is denoted in the electronic hospital information system and hence visible to all physicians and nurses involved in the care of this patient. All adverse events will be reported immediately to the research coordinator and principle investigator. The principal investigator will report suspected unexpected serious adverse reactions to the federal health authorities.

### Sample size calculation

The coefficient of variation (CV) in postoperative morphine consumption was assumed to equal 0.7 and derived from own data as reported by Dewinter et al. To have 80% power to show a 30% reduction of the 24-h consumption in the ropi group versus the placebo group using a two-sided test for a ratio of means (with alpha = 5%), at least 51 patients per group are needed (i.e., 102 patients in total). One interim analysis for futility is planned after accrual of 50% of the participants. To preserve the desired power level, 106 subjects in total are required, based on the O’Brien-Fleming-type error spending function and applying a non-binding boundary to have the flexibility to continue the trial when the test statistic falls in the acceptance region at the interim analysis [16]. The total number of subjects will be increased to 128 to have also at least 80% power in a secondary analysis at the final stage to detect an effect of ropivacaine within the subgroup of patients with a BMI ≤ 30 (this group is expected to constitute 80% of the total sample). In order to compensate for possible dropouts, we will increase the number of patients by 15% to include 150 patients in total. The sample size calculation was performed using East 6 (East 6, Statistical software for the design, simulation, and monitoring of clinical trials, Cytel Inc., Cambridge, MA: https://www.cytel.com/software/east).

### Data analysis

For the primary outcome, a two-sided test for the ratio of means will be used to compare the 24-h cumulative morphine intake between both groups. The test will be based on a linear model on log-transformed values with group and the stratification variable (BMI ≤ 25) as factor. A 95% confidence interval for the ratio (of the geometric means) will be reported. A stratified Mann-Whitney *U* test will be used to test the robustness of the conclusion if the log-transformed data showed a departure from normality based on the Shapiro-Wilk *W* test statistic. As a secondary analysis, the same approach will be applied in the subgroup of patients with a BMI ≤ 30. As a further exploratory analysis, it will be verified if the effect of ropivacaine depends on BMI by adding the interaction between group and BMI into the model (depending on the distribution of BMI, treating BMI as a continuous variable or categorizing BMI into more than two groups).

After inclusion of 50% of the subjects, a non-binding interim analysis will be performed based on the O’Brien-Fleming-type error spending function, accepting the null hypothesis when the *Z*-statistic ≤ 0.55 (*p* value ≥ 0.291). This corresponds to a conditional power (to show a difference) equal to 0.047 and 0.427 when the remaining data are sampled from a population defined by the parameters under the null and the alternative hypothesis, respectively.

Secondary outcomes will be compared using Fisher’s exact test in case of proportion measurements, and Mann-Whitney *U* tests will be used when the data is measured on a ratio or ordinal level. A linear model for longitudinal measurements (with the selection of the covariance structure based on the Akaike Information Criterion) will be used for variables that are measured over time (NRS for pain). The number of times morphine IV PCA is requested will be analyzed using a model for count data (Poisson or negative binomial model, depending on presence of overdispersion). Incidence of local anesthetic systemic toxicity (LAST) will be compared using Fisher’s exact test. A Mann-Whitney *U* test will be used for the maximum number of dermatomes, separately at two time points (after the first and after the last dose). The postoperative evolution of the incidence of nausea and vomiting will be evaluated with a logistic regression model with generalized estimating equations (GEE). Fisher’s exact test will be used for the comparison of the presence of “ever PONV” during the postoperative follow-up, as well as for each of the early safety endpoints at 30 days (serious adverse events).

All analyses will be based on the intention-to-treat (ITT) principle, and a *p* value smaller than 0.05 will be considered significant.

Analyses will be performed using SAS software, version 9.4 of the SAS System for Windows.

A co-investigator or a study nurse will review completed case record forms for completeness and correctness before digitalization and statistical analysis. Case record forms will be completed from data drawn from the source documents and the electronic hospital information system. Missing data is not expected as all clinical data are mandatorily collected in the electronic hospital information system. Data will be coded and analyzed in line with the intention-to-treat principle.

## Discussion

Local regional anesthesia plays an important role in multimodal pain management. In the last decade, abdominal wall blocks such as the TAP block have been shown to be efficient in the control of postoperative pain in patients undergoing laparoscopic colorectal surgery [17–19]. However, the number of trials with negative outcomes is increasing [20, 21]. Most of the abdominal wall blocks, like TAP and fascia transversalis block, probably only affect somatic wall pain. Recently, we performed a trial on the efficacy of the fascia transversalis block (named QLB1 at that time and published under that name) in laparoscopic colorectal surgery in which we were unable to find any significant improvement with respect to postoperative pain and opioid requirements [5]. To treat visceral pain, a spread of the local anesthetic to the paravertebral space is mandatory. When performing a TQL block, the spread of the local anesthetic is expected to be ideally between psoas major muscle and quadratus lumborum muscle, where the ventral rami of the spinal nerve pass by at the dorsal side of the quadratus lumborum muscle.

Until now, the most effective analgesic strategy in these patients especially in an enhanced recovery program is still unknown. Recognizing the importance of procedure-specific pain management, we aim to investigate whether a TQL block delivers superior pain control in comparison to PCIA with morphine alone.

### Safety issues

The interventional treatment will be performed under hemodynamic monitoring in a fully equipped preoperative block room. Risk of local anesthetic systemic toxicity will be minimized by ultrasound guidance and needle aspiration before injection [22]. Patients are admitted to the PACU following surgery. A dedicated nurse will follow the patients’ vital signs, and a computer-generated early warning system is continuously monitoring these vital signs.

The TQL block has been shown to be safe in numerous reports. Large doses of local anesthetics carry the potential risk of local anesthetic systemic toxicity and can affect the cardiovascular system and central nervous system [23]. As a safety precaution, patients will be continuously monitored in the block room and in the operating theater with pulse oximetry, electrocardiogram, and invasive blood pressure, until at least 1 h after the last administration of the trial medication according to the guidelines of the ASRA [24]. In case of symptoms of LAST, code-break is allowed to start adequate treatment. Local anesthetic delivery will be stopped, airway secured, and cardiovascular resuscitation initiated. Early treatment with lipid emulsion 20% will be initiated [23].

Also, the inclusion of each patient into the trail is denoted in the electronic hospital information system. Hence, this is visible to all physicians and nurses involved in the patients’ care. All adverse events will be reported immediately to the research coordinator and principal investigator. The latter will report suspected unexpected serious adverse event to the federal health.

### Advantages for the participating patients

There is no guarantee that the use of TQL block with local anesthetic ropivacaine will provide a benefit to the participating patient.

### Trial status

Patient recruitment will start in October 2019. The predicted study completion date is December 2020. Protocol version SC 05 – 19-07-2019.

## Data Availability

The datasets generated and analyzed during the current study are not publicly available due to regulatory restrictions (Directive 95/46/EC and Belgian Law of December 8, 1992, on the Protection of the Privacy in relation to the Processing of Personal Data). Data are however available from the authors upon reasonable request and with permission of the ethics committee.

## Abbreviations

TAP: Transversus abdominis plane
TQL: Transmuscular quadratus lumborum
PONV: Postoperative nausea and vomiting
PACU: Postoperative anesthesia care unit
PCIA: Patient-controlled intravenous anesthesia
PCEA: Patient-controlled epidural anesthesia
EA: Epidural analgesia
BMI: Body mass index
ITT: Intention-to-treat
GEE: Generalized estimating equations
LAST: Local anesthetic systemic toxicity
IV: Intravenous
NRS: Numeric rating scale
TOP: Transverse oblique paramedian
CV: Coefficient of variation
ERAS: Enhanced Recovery After Surgery
